# Synthesis of Bis-Terpyridine-Based Metallopolymers and the Thermoelectric Properties of Their Single Walled Carbon Nanotube Composites

**DOI:** 10.3390/molecules26092560

**Published:** 2021-04-28

**Authors:** Jiahua Li, Zeling Guo, Linli Xu, Wai-Yeung Wong

**Affiliations:** 1 Department of Applied Biology and Chemical Technology and Research Institute for Smart Energy, The Hong Kong Polytechnic University, Hung Hom, Kowloon, Hong Kong, China; koopa.l.li@connect.polyu.hk (J.L.); zeling.guo@connect.polyu.hk (Z.G.); linli.xu@polyu.edu.hk (L.X.); 2 The Hong Kong Polytechnic University Shenzhen Research Institute, Shenzhen 518057, China

**Keywords:** thermoelectric, metallopolymer, terpyridine, composite

## Abstract

Although the organic and the conventional inorganic thermoelectric (TE) materials have been extensively developed in recent years, the number of cases involving conducting metallopolymers is still quite limited. In view of the versatile coordination capability of the terpyridine fraction and the electron-rich nature of the 3,4-ethylenedioxythiophene moiety, a bis-terpyridine-featured ligand was designed, and a series of metallopolymers were then synthesized. Upon the addition of single-walled carbon nanotube (SWCNT), the TE properties of the resulting metallopolymer-SWCNT composite films were investigated. It was found that metal centres played an important role in affecting the morphology of the thin films, which was a key factor that determined the TE performances of the composites. Additionally, the energy levels of the metallopolymers were feasibly tuned by selecting different metal centres. With the combined effects of a uniform and condensed surface and an optimized band structure, the highest power factor was achieved by the Cu(II)-containing metallopolymer-SWCNT composite at the doping ratio of 75%, which reached 38.3 μW·m^−1^·K^−2^.

## 1. Introduction

As a green approach to convert heat into electrical energy, thermoelectric (TE) materials are showing their promising prospect in both macro-scaled and mini-scaled applications, such as power generation, health monitors, etc. [1,2], and thus have received tremendous attention. The TE performance of a material is assessed by its figure-of-merit (*ZT*): *ZT* = *S*^2^*σT*/*κ*, where *S* is the Seebeck coefficient (V·K^−1^), *σ* is the electrical conductivity (S·m^−1^), *T* is the absolute temperature (K), and *κ* is the thermal conductivity (W·m^−1^·K^−1^) [2,3]. Apparently, materials exhibiting high electrical conductivity, high Seebeck coefficient but low thermal conductivity are expected. In this respect, conventional inorganic materials do a great job as they are always demonstrated to show high *ZT* values exceeding 1.0. For example, via the hot pressing technique, nanocrystalline BiSbTe bulk materials were synthesized from its nanopowders, achieving a peak *ZT* of 1.4 at 100 °C [4]. By controlling the doping ratio of iodine, the n-type semiconductor PbTe_1−*x*_I*_x_* gave the *ZT* value up to 1.4 from 700 K to 800 K [5]. In 2020, Zhu et al. developed a bismuth antimony chalcogenide material with the highest *ZT* value reaching ca. 1.4 within a temperature range from 300 K to 575 K [6].

Despite the satisfactory energy conversion efficiency, these inorganic materials bear inevitable shortcomings, including the use of heavy metals and rare elements, their hard and fragile characters, and high performances only at high temperature [7,8,9]. In contrast, by virtue of good flexibility, light weight, diverse chemical structures, and the ease of processing, a number of organic TE materials have been synthesized and studied. In light of the extremely low thermal conductivity (generally below 1 W·m^−1^·K^−1^) [10], another parameter—power factor (*PF*)—is useful to characterize the TE performance of the organic materials as: *PF* = *S*^2^*σ* [7,9,10]. As the most successful instance, poly(3,4-ethylenedioxythiophene):poly(styrenensulfonate) (PEDOT:PSS) has been extensively studied for years, not only due to its high TE performance (sometimes comparable to the inorganics), but it also possesses some attractive features including the realised processibility in aqueous media and high thermal stability [6,11]. By adjusting the stacking structure of PEDOT:PSS, the prepared thin film gave a high *PF* up to 330.597 μW·m^−1^, and a device was made to recycle the heat in the form of solar energy [12]. Besides, some electron-rich conducting polymers including polypyrroles [13,14,15,16], polythiophenes, and their composites [17,18] also exhibited their potential in TE applications.

Emerging as a new class of TE materials, metallopolymer-based TE materials bring the advantages of metallation to pure organic polymers [9]. The involvement of appropriate transition metal ions could increase the probability to provide active species during the electrical conducting process [19,20,21], tune the energy levels through d-π conjugation feasibly [19,22], generate various molecular geometries according to the coordinating properties of the metal centres and lower the thermal conductivity [9]. In this regard, Zhu et al. successfully developed a series of 1,1,2,2-ethenetetrathiolate-based metallopolymers [poly(M(ett)] with tunable TE performances when different metal centres or processing methods were adopted [23,24,25]. For example, poly[Na*_x_*(Ni-ett)] and poly[K*_x_*(Ni-ett)] were demonstrated to be n-type semiconductors with their room-temperature (RT) *PF* of around 26 μW·m^−1^·K^−2^, but poly[Cu*_x_*(Cu-ett)] was a p-type TE material which produced the RT *PF* of 38.6 μW·m^−1^·K^−2^ [23]. Other representative cases mainly included 2,3,6,7,10,11-hexaiminotriphenylene- [26,27] and porphyrin-based 2D framework materials [28,29] and metalated poly(Schiff base)s [30,31]. However, the number of metallopolymer-derived TE materials is still quite limited.

In light of the versatile coordination capability [32,33,34,35,36], the terpyridine moiety always appears as a popular fragment in coordination chemistry, and its related materials were found to exhibit interesting properties in a wide range of applications. For example, in 2016, Liang et al. synthesized a series of bis(terpyridine)-based Fe(II) complexes which displayed rapid and reversible electrochromic behaviour between blue and pale yellow upon repeated redox process [37]. By employing a hexylthiophene-modified terpyridine as the ligand and Ru(II) as the metal centre, two ruthenium-containing sensitizers for solar cells have been developed and improved molar absorption coefficients were observed [38]. Additionally, a metal-organic framework based on Eu(III) and terpyridine was proved to be a highly selective and sensitive probe for Fe^3+^ in aqueous solution and even in biological systems [39]. Moreover, Elgrishi found that the reduction of proton and CO_2_ could be feasibly realised under the catalysis of several cobalt-terpyridine coordination compounds [40]. However, the application of terpyridine-based materials towards TEs is still rarely seen.

**Figure 1:**
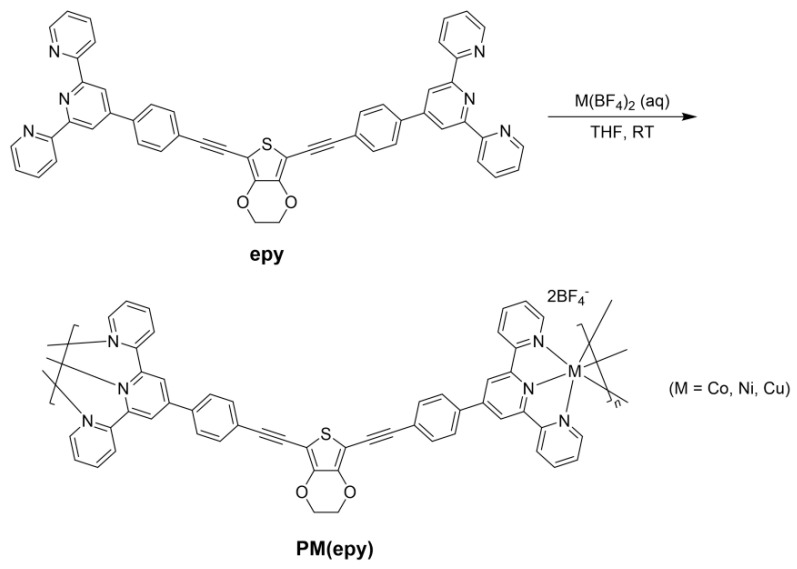
Synthetic route of **PM(epy)**.

In this work, a bis(terpyridine)-based new ligand was designed. The EDOT fragment was selected as the bridge to link the two terminal terpyridine chelating sites, due to its electron-rich property as well as the potential capability to enhance the TE performance [41]. Through the coordination with various transition metal ions (Co^2+^, Ni^2+^ and Cu^2+^), a series of terpyridine-based metallopolymers were prepared (Figure 1). Then, by blending with single-walled carbon nanotubes (SWCNTs), the TE properties of the resulting composites were measured at different doping ratios (*f*_C_, *f*_C_ = *m*_SWCNT_/*m*_composite_). The factors that affected the TE performance are discussed in detail from the perspectives of the morphology evolution and the role played by metal centres were highlighted. It is the first time that the TE performance of the terpyridine-based metallopolymer–SWCNT composite materials was assessed systematically.

## 2. Materials and Methods

### 2.1. Materials

Co(BF_4_)_2_·6H_2_O and Cu(BF_4_)_2_·6H_2_O were purchased from Energy Chemical, and Ni(BF_4_)_2_·6H_2_O was purchased from Alfa Aesar. SWCNTs (purity > 95%), with diameters of 1–2 nm and lengths of 5–30 μm, were purchased from XFNANO. 1,2-Dichloroethane was purchased from TCI, and other organic solvents were purchased from Alfa Aesar. Flat glass sheets with a dimension of 15 mm × 15 mm × 1 mm (length × width × height) were cleaned and used as substrates for the film deposition. As a typical cleaning procedure, the glass sheets were fully immersed and sonicated in the aqueous solutions of 5% hydrochloric acid and 5% sodium carbonate successively, washed with deionized water before and after each sonication, and finally rinsed with absolute ethanol thoroughly before drying *in vacuo*.

### 2.2. Synthesis

#### 2.2.1. Preparation of the Metallopolymer **PM(epy)**

The detailed synthetic route is shown in Figure S1. As a general procedure, 19 mg **epy** (24 μmol) was dissolved in 10 mL THF under nitrogen. With vigorous stirring, a solution of 24 μmol M(BF_4_)_2_ in a mixed solvent of 0.5 mL deionized water and 2 mL MeOH was added into the ligand solution in a dropwise manner. The reaction was maintained at RT for 1 day, after which the precipitate was collected by filtration, washed thoroughly with deionized water and THF, and dried in a vacuum oven. Pure **PCo(epy)**, **PNi(epy)**, and **PCu(epy)** were furnished as a red powder, a dark yellow powder, and a brown powder, respectively.

#### 2.2.2. Preparation of **PM(epy)**-SWCNT Composite Films

In a round-bottomed flask, 50 mg SWCNT and 50 mL 1,2-dichloroethane were mixed. Then, the flask was immersed in an ice bath at 0 °C and the mixture was dispersed with an ultrasonic homogenizer at a power of 95 W for 1.5 h. To a glass vial containing a specific amount of the metallopolymer powder, 1.0 mL of the formed SWCNT dispersion was added. Then, the vials were sealed and sonicated at 0 °C for 2 h. After that, gel-like mixtures were formed and dropped onto clean glass substrates. Qualified thin composite films were obtained after solvent evaporation and their thickness was measured before the measurements of the TE properties.

### 2.3. Characterization

The nuclear magnetic resonance (NMR) spectra were acquired on a Varian VNMRS 400 spectrometer or a Bruker AVANCE III 400 spectrometer. ^1^H NMR spectra were quoted relative to the internal reference tetramethylsilane (TMS, *δ* = 0.00 ppm). For ^13^C NMR acquisitions, the spectra were referenced to the recommended values [42] of the deuterated solvent signals: *δ*(*d*_6_-acetone) = 29.84 ppm, *δ*(CDCl_3_) = 77.16 ppm, and *δ*(*d*_8_-THF) = 25.31 ppm. Matrix-assisted laser desorption/ ionization-time of flight (MALDI-TOF) mass spectra were acquired on a Bruker UltrafleXtreme MALDI-TOF mass spectrometer with *α*-cyano-4-hydroxycinnamic acid or 2,5-dihydroxybenzoic acid employed as the matrix. Samples for Fourier-transform infrared (FTIR) spectroscopy were prepared by using the KBr pellets and the spectra were measured on an Agilent Cary 670 spectrometer. Raman spectra were collected on a Renishaw Micro-Raman microscope, under the irradiation of a laser at 785 nm. Scanning electron microscopic (SEM) images were captured on a JEOL JSM-6490 electron microscope. Transmission electron microscopic (TEM) images were obtained on a JEOL JEM-2100F model. Elemental analysis was tested on a Vario EL Cube elemental analyser. UV-Vis reflection spectra of powdered samples were collected on a Varian Cary 4000 spectrophotometer with the integrating sphere accessories. The UV-Vis-NIR absorption spectrum was measured on a Shimadzu UV-3600 Plus spectrometer. The X-ray photoelectron spectra (XPS) were acquired on a Thermo Scientific Nexsa system. Each survey spectrum was accumulated for 5 times and each high-resolution spectrum was acquired with 20 scans. The thickness of the thin films was measured on a Bruker DektakXT Surface optical profiler equipped with a diamond-tipped stylus showing a radius of 2.5 µm.

The TE properties of the thin films were tested on a JouleYacht MRS-3RT TE Testing System at RT. The glass substrate with the thin film was fixed in the sample chamber where light was avoided. The four-point method was applied to measure the electrical conductivity. For the tests of Seebeck coefficient, the temperature gradient was generated between two thermocouples where one maintained the temperature of the cold terminal at around RT and the other one heated the films up gradually. The highest temperature of the hot end was confined within 60 °C and the entire heating process took ca. 60 s. The whole course was controlled automatically by the system and the data were recorded in real time. Upon each completion of the Seebeck coefficient measurement, the heating process stopped immediately and the thin film was allowed to cool to RT naturally.

## 3. Results and Discussion

### 3.1. Chemical Structure Determination

**Figure 2:**
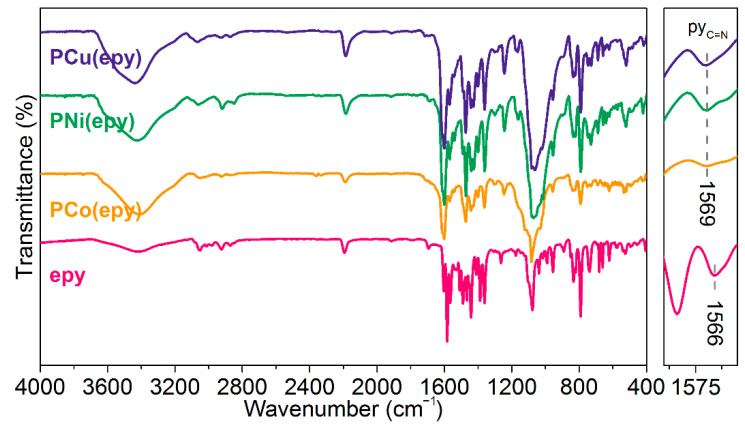
FTIR spectra of **PM(epy)**. Expansion (right): the spectral region involving the absorption of C=N bond from the pyridine ring.

Due to the highly conjugated and rigid molecular structures, all these metallopolymers were insoluble in common organic solvents. Consequently, their nuclear magnetic resonance spectra were not obtained. As a workaround, FTIR spectroscopy was used to determine the chemical structures of the products. As displayed in Figure 2, the absorption bands within the spectral region from 3200 cm^−1^ to 2800 cm^−1^ were assigned to the stretching vibration of C-H bond [43]. The peaks shown at 2186 cm^−1^ depict the presence of the internal C≡C bond [43]. After the metal coordination, the absorption bands at around 1078 cm^−1^ were broadened, probably due to the combination of the signals from B-F bonds [44]. Particularly, the absorption peak corresponding to the C=N stretching vibration of the pyridine ring [34] from **epy** at 1566 cm^−1^ shifted to the long wavelength region, which implied the successful coordination of the metal ions.

### 3.2. Electrochemical Properties

**Figure 3:**
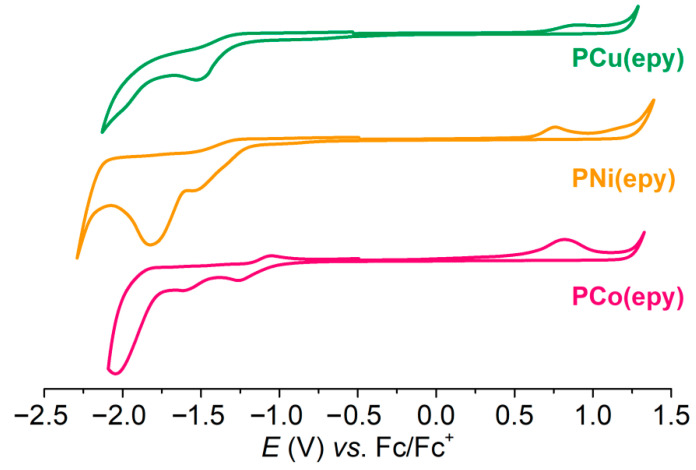
Cyclic voltammograms of **PCo(epy)**, **PNi(epy)**, and **PCu(epy)** in degassed CH_2_Cl_2_.

The electrochemical properties of **PM(epy)** were investigated by cyclic voltammetry (CV). As shown in Figure 3, all metallopolymers exhibited both oxidative and reductive waves, implying their capabilities to convey electrons and holes [45]. At 0.45 V, an irreversible oxidation of the organic backbone was detected for all metallopolymers, from which the highest occupied molecular orbital (HOMO) or singly occupied molecular orbital (SOMO) energy values were calculated and listed in Table 1. It can be seen that the incorporation of these metal ions hardly affected the positions of the HOMO or SOMO levels. **PNi(epy)** exhibited an additional wave at ca. 0.97 V, which may be ascribed to the oxidative process from Ni(II) to Ni(III) [20]. At the reduction region, a characteristic redox-reversible process was detected at −1.16 V from **PCo(epy)**, which revealed the electrochemical conversion between Co(II) and Co(I) [46,47,48]. By comparison, the electrochemical process occurring on the metal centre of **PCu(epy)** was not found, which implied the redox-inactive property of Cu(II) [20].

### 3.3. Morphology Studies

**Figure 4:**
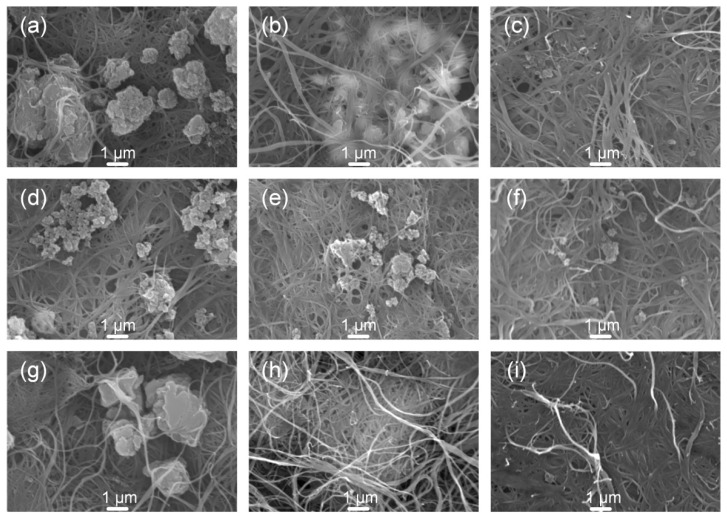
SEM images of the (**a**–**c**) **PCo(epy)**-SWCNT, (**d**–**f**) **PNi(epy)**-SWCNT, and (**g**–**i**) **PCu(epy)**-SWCNT composites films with the doping ratio (*f*_C_) of 15% (left column), 60% (middle column), and 90% (right column).

The morphology of the prepared composite thin films was studied under an SEM. As revealed in Figure 4, the morphology change of the thin films heavily depended on the SWCNT content. At *f*_C_ = 15%, serious macro-phase separation was observed. SWCNTs formed condensed layered structures with large metallopolymer aggregates sitting atop or sandwiched between adjacent layers. A closer observation found that the aggregated **PNi(epy)** particles are of smaller size than other two analogues (Figure 4d), suggesting an improved contact area with SWCNTs. **PCu(epy)** formed a layer-by-layer structure with smooth surface (Figure 4g), which was in favour of establishing strong interfacial interactions with the SWCNTs. At the medium doping level (*f*_C_ = 60%), much better dispersed composites were developed, except for **PCo(epy)**-SWCNT where the aggregation of the metallopolymer was not effectively alleviated (Figure 4b). By contrast, the **PCu(epy)**-SWCNT composite was highlighted by its uniform morphology (Figure 4h). All metallopolymer particles were perfectly dispersed and coated on the SWCNT network evenly. With the CNT content increased up to 90%, a homogeneous surface was evolved. A condensed and inter-connected CNT network was observed and the inter-layered interaction was significantly enhanced with only a few **PCo(epy)** and **PNi(epy)** particles appeared, which provided an efficient charge carrier transporting system.

### 3.4. Raman Spectral Analysis of the **PM(epy)**-SWCNT Composites

**Figure 5:**
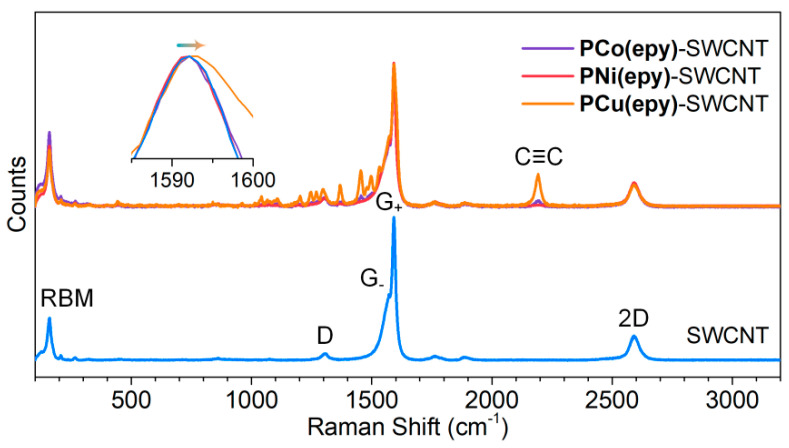
Raman spectra of pure SWCNTs and the **PM(epy)**-SWCNT composites at *f*_C_ = 60%.

The gradual evolution of the morphology possibly implied the effective establishment of the interactions between the metallopolymers and SWCNTs. Here, Raman spectra were acquired for verification. As shown in Figure 5, a series of characteristic absorption peaks were detected from the Raman spectrum of the pure SWCNT thin film, including a radial breathing mode (RBM) at 161 cm^−1^, a D-band from sp^3^-hybridized carbon atoms at 1306 cm^−1^, a G-band from sp^2^-hybridized carbon atoms (involving a G_-_ band at 1571 cm^−1^ and a G_+_ band at 1592 cm^−1^), and a 2D band at 2591 cm^−1^ [49]. After doping with **PM(epy)**, a slight spectral shift of the G_+_ band was found, especially for **PCu(epy)**-SWCNT composite whose peak shifted to the long wavelength region the most significantly, which may benefit from the increased contact area. This phenomenon revealed the presence of the interfacial interactions between the metallopolymers and the SWCNTs. Besides, for the composites, the emergence of the new peak at 2192 cm^−1^ corresponded to the stretching vibration of the C≡C bond. The presence of additional peaks from **PCu(epy)** may imply that the energy gap of the metallopolymer matched well with the energy of the incident laser.

### 3.5. TE Performances

**Figure 6:**
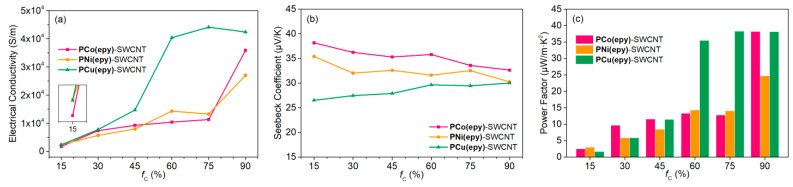
(**a**) Electrical conductivity, (**b**) Seebeck coefficient, and (**c**) *PF* of the **PM(epy)**-SWCNT composites at various *f*_C_.

**Figure 7:**
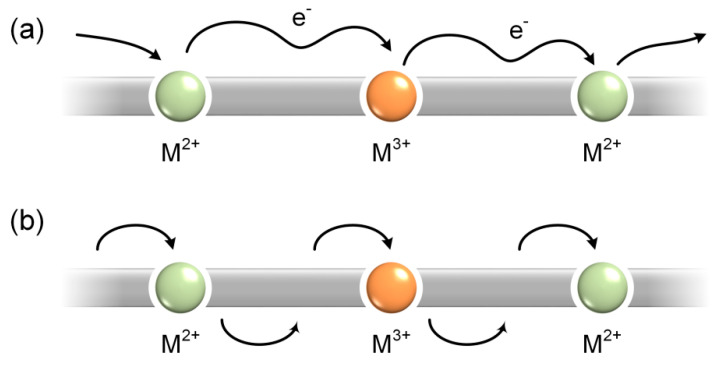
The charge carrier transmission in Wolf type III conducting metallopolymers via (**a**) superexchange or (**b**) hopping behaviours.

The electrical conductivities and the Seebeck coefficients of the prepared composites were assessed on a JouleYacht MRS-3RT TE testing system at RT. From Figure 6a, the increasing amount of SWCNT helped to boost the electrical conductivity of the composite remarkably. At *f*_C_ = 15%, **PNi(epy)**-SWCNT and **PCu(epy)**-SWCNT showed almost the same conductivity, which was 60% higher than that of the **PCo(epy)**-SWCNT composite. As for **PNi(epy)**, the CV curves displayed that the oxidation wave of the metal centre was quite close to that of the organic backbone, which may imply a redox-matched scenario. Judging from the molecular structures, all these metallopolymers are Wolf type III conducting polymers where metal centres appear in the polymer main chain and act as linkers [50]. In a typical conducting process, the oxidized organic backbone was doped and became a highly conductive bridge or a hopping station between any adjacent two metal centres at different oxidation states (Ni^2+^/Ni^3+^). The resulting inter-chain interactions and intra-chain charge transfer in superexchange (Figure 7a) and charge hopping (Figure 7b) fashions together account for the high electrical conductivity [19,20]. In the case of **PCu(epy)**-SWCNT, although Cu^2+^ was considered redox-inactive and the composite should have suffered from low conductivity, a high conductivity was detected unexpectedly. The possible reasons could be due to the flat surfaces of **PCu(epy)** particles, the resulting stronger interactions with the SWCNT networks and the paramagnetic property of Cu^2+^ [51]. By contrast, large aggregates of **PCo(epy)** not only enlarged the distance between adjacent SWCNT networks, but also decreased the contact area. The increment of the doping ratio brought about a significant enhancement on the electrical conductivities of all composites, which benefited from the gradual formation of a uniform composite surface and increased charge carrier concentration. Notably, **PCu(epy)**-SWCNT always kept the highest value at all doping ratios, with the top value reaching 4.41 × 10^4^ S·m^−1^.

The dependence of Seebeck coefficient on *f*_C_ was shown in Figure 6b. As expected, all samples gave positive *S* values, suggesting that holes were the main charge carriers and these composites were p-type conductors. When more SWCNT were introduced, the Seebeck coefficients of **PCo(epy)**-SWCNT and **PNi(epy)**-SWCNT dropped, which was caused by the inversed relationship between electrical conductivity and Seebeck coefficient [3]. However, the *S* value of the **PCu(epy)**-SWCNT composites climbed up slightly. Such an interesting phenomenon probably stemmed from the energy filtering effect at the **PCu(epy)**-SWCNT interfaces. At the crystallite boundaries, such an effect filtered off most low-energy charge carriers and only those with high energy were allowed to transmit [52,53,54]. In view of the better developed interfacial interactions, the energy filtering effect played a leading role in determining the Seebeck coefficient, and therefore gradually elevated *S* values were recorded at high *f*_C_.

The *PF*s of the composite thin films were shown in Figure 6c, which exhibited a similar variation trend to the electrical conductivity. The highest *PF* of 38.29 μW·m^−1^·K^−2^ was achieved by **PCu(epy)**-SWCNT at *f*_C_ = 75%. In summary, the TE properties of the **PM(epy)**-SWCNT composites at *f*_C_ = 75% are summarized in Table 2.

### 3.6. Energy Band Analysis

**Figure 8:**
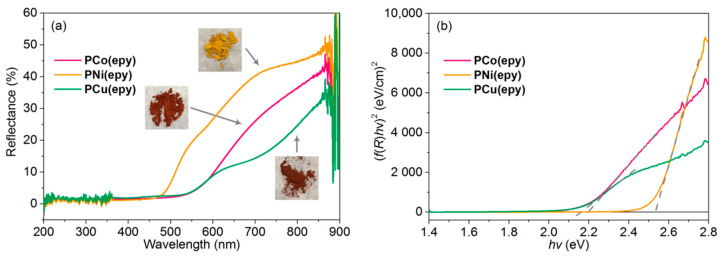
(**a**) UV-Vis diffused reflection spectra of neat **PM(epy)** powders. Inset: the photos of each sample taken under daylight. (**b**) The Kubelka–Monk function versus energy plots of the metallopolymers.

The solid state UV-Vis diffused reflection spectra (Figure 8a) of neat **PM(epy)** were acquired to further investigate the factors that affect the electrical conductivity from the perspective of band structure. For the convenience of analysis, the reflection spectra were transformed into the Kubelka–Monk function—photon energy plot (Figure 8b). The optical energy gaps of **PCo(epy)**, **PNi(epy)**, and **PCu(epu)** were measured to be 2.20 eV, 2.53 eV, and 2.16 eV, respectively, suggesting that **PCu(epy)** possessed the narrowest optical band gap and the smallest activation energy. Therefore, **PCu(epy)** was expected to afford the highest electrical conductivity. Besides, as the HOMO level of SWCNT was positioned at −5.05 eV [55,56], **PCu(epy)** also gave the narrowest energy gap between the SOMO level of the metallopolymer and the HOMO level of the SWCNT, which was in favour of the hole transport [55] and thus rendered **PCu(epy)**-SWCNT the most conductive composites in the metallopolymer-SWCNT blending system at almost all doping ratios. As for the **PNi(epy)**-SWCNT composites, although the redox-matching effect was present, the obvious phase separation morphology of the composite films and the wide optical energy gap of **PNi(epy)** together diminished the electrical conductivity.

## 4. Conclusions

In summary, three terpyridine-based metallopolymers were synthesized and blended with SWCNT to assess the TE performances of the resulting composite films. CV curves of the neat metallopolymers indicated that the organic backbone was oxidized at ca. 0.45 V and **PNi(epy)** displayed an additional oxidation wave corresponding to the conversion from Ni(II) to Ni(III) at 0.97 V. Morphology studies showed uniform thin films were gradually formed with an elevated content of SWCNTs, which promoted high electrical conductivities. Raman spectra revealed the presence of the interfacial interactions between the metallopolymers and SWCNTs. TE tests indicated that the electrical conductivity was remarkably enhanced when more SWCNTs were introduced. Particularly, **PCu(epy)**-SWCNT composites always possessed the highest values compared with other two analogues (i.e., **PCo(epy)**-SWCNT and **PNi(epy)**-SWCNT), which was benefited from the well dispersed morphology and the optimized band structures. Among all the prepared composite thin films, the highest *PF* was achieved by the **PCu(epy)**-SWCNT composite at *f*_C_ = 75%, reaching a value of 38.3 μW·m^−^^1^·K^−2^. This research revealed a promising application prospect of terpyridine-based metallopolymer materials towards TEs, and the metallation process was demonstrated to be a critical but convenient approach to tune the TE properties of these materials.

## Figures and Tables

**Table 1:** The HOMO or SOMO energies of **PM(epy)**.

Metallopolymers	HOMO or SOMO (eV)
**PCo(epy)**	−5.00
**PNi(epy)**	−5.01
**PCu(epy)**	−5.06

**Table 2:** The TE properties of the **PM(epy)**-SWCNT composites at *f*_C_ = 75%.

Composites	*σ* (S·m^−1^)	*S* (μV·K^−1^)	*PF* (μW·m^−1^·K^−2^)
**PCo(epy)**-SWCNT	1.13 × 10^4^	33.6	12.8
**PNi(epy)**-SWCNT	1.33 × 10^4^	32.5	14.1
**PCu(epy)**-SWCNT	4.41 × 10^4^	29.5	38.3

## Data Availability

Data are contained within the article or the Supplementary Materials.

## Supplementary Materials

The following are available online, Figure S1: Detailed experimental procedures; Figure S2: XPS of the metallopolymers; Figure S3: NMR spectra and mass spectra of the intermediates; Figures S4–S7: UV-Vis-NIR absorption spectrum of the SWCNT dispersion; Figure S8: TEM image of the SWCNT bundles.

## Sample Availability

Samples of the compounds **1**, **2** (see Figure S1 in Supporting Materials), **epy**, and **PM(epy)** are available from the authors.
