# Supplementary material for: Synthesis of Bis-Terpyridine-Based Metallopolymers and the Thermoelectric Properties of Their Single Walled Carbon Nanotube Composites
Source: Molecules. 2021 Apr 28;26(9):2560. doi: 10.3390/molecules26092560 (PMC8124700; doi:10.3390/molecules26092560)
Supplement: Supplementary file 1 [file molecules-26-02560-s001.zip › molecules-1152430-supplementary.pdf]

molecules

Supplementary Materials

Synthesis of Bis-Terpyridine-Based Metallopolymers and the Thermoelectric Properties of Their Single Walled Carbon Nanotube Composites

Jiahua Li [1], Zeling Guo [1], Linli Xu [1,2] and Wai-Yeung Wong [1,2,*]

[1] Department of Applied Biology and Chemical Technology and Research Institute for Smart Energy, The Hong Kong Polytechnic University, Hung Hom, Kowloon, Hong Kong, China
[2] The Hong Kong Polytechnic University Shenzhen Research Institute, Shenzhen 518057, China
* Correspondence: wai-yeung.wong@polyu.edu.hk

1. Experimental Procedures

1.1. General Information

3,4-Ethylenedioxythiophene, *N*-bromosuccinimide (NBS), CuI and 2-ethoxyethanol were purchased from Alfa Aesar. 4-Ethynylbenzaldehyde was synthesized according to the previous report. Diisopropylamine and 1,4-dioxane were purchased from Acros. Dioxane was distilled over sodium metal / benzophenone under nitrogen prior to use. 2-Acetylpyridine was purchased from Sigma-Aldrich. SWCNTs (diameter: 1–2 nm, length: 5–30 μm, purity > 95%) were purchased from XFNANO. Other chemicals were purchased from Energy Chemical and were used as received unless otherwise stated.

1.2. Synthesis

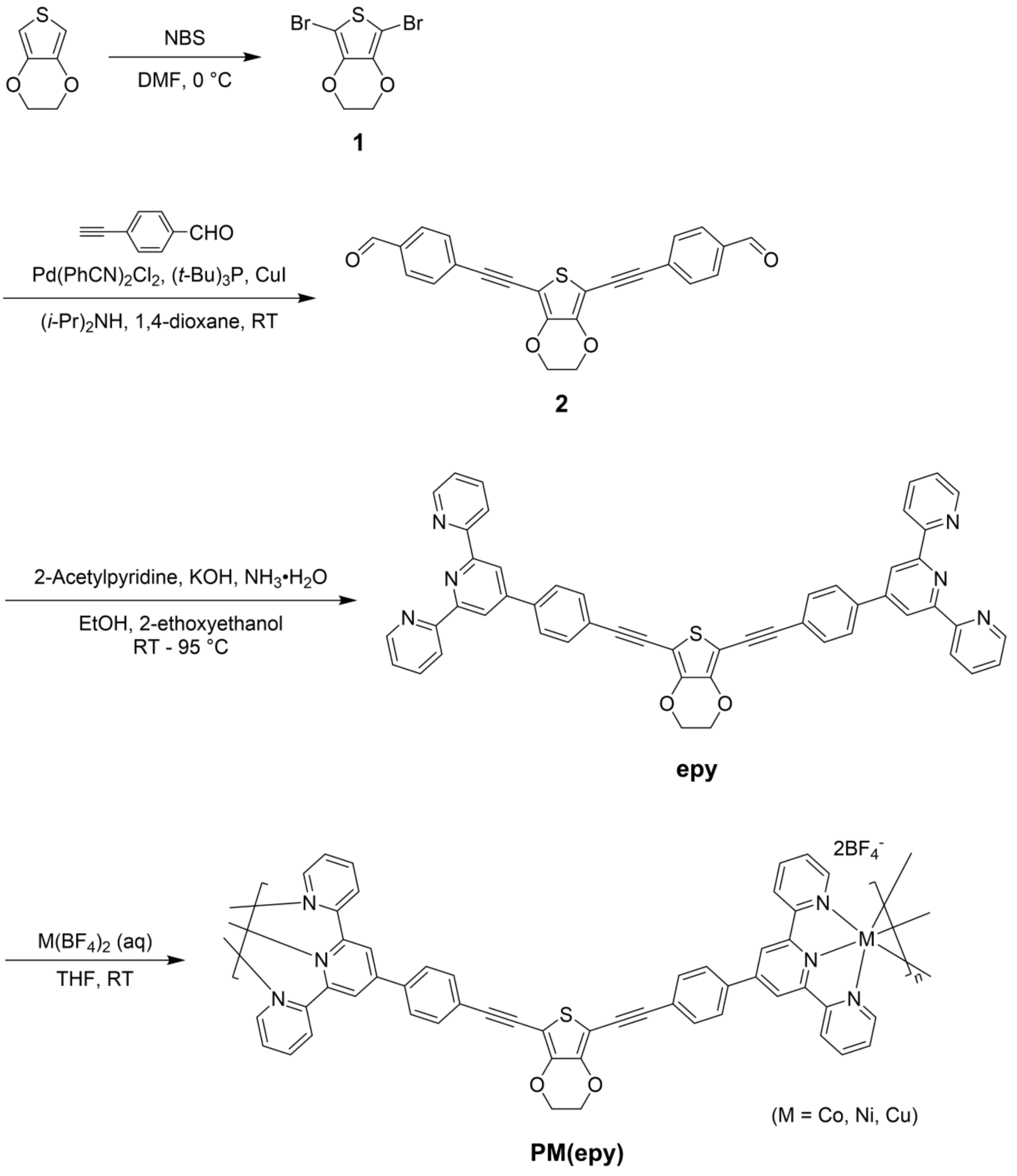

Figure S1. Synthetic route of **PM(epy)** (M = Co, Ni, Cu).

1. In a 250 mL round bottom flask, 1.42 g 3,4-ethylenedioxythiophene (1.0 equiv., 10 mmol) was diluted with 30 mL DMF under the protection of nitrogen. The mixture was stirred at 0 °C for 15 min, after which a solution of 3.78 g NBS (2.1 equiv., 21 mmol) in 25 mL DMF was dropped into the flask slowly. Upon completion, the reaction mixture was kept at room temperature (RT) for 2 h. Then 100 mL deionized water was poured into the flask in one go to quench the reaction. The precipitate was filtered, washed with deionized water (40 mL × 3) and 10 mL cold ethanol successively, and dried in *vacuo*, which afforded the pure product as a white powder in 95% yield. ^{1}H NMR (400 MHz, $CDCl_3$) δ 4.13 (s, 4H). ^{13}C NMR (100 MHz, $CDCl_3$) δ 139.3, 85.1, 64.6.

2. This compound was prepared by following Fu's protocol [1] with slight modifications. To a Schlenk tube, 1.50 g compound **1** (1.0 equiv., 5.0 mmol), 0.115 g $Pd(PhCN)_2Cl_2$ (6%, 0.3 mmol) and 0.038 mg CuI (4%, 0.2 mmol) were added. The tube was degassed and refilled with N_2 for 5 times. After that, 10 mL freshly distilled

1,4-dioxane, 0.60 mmol (t-Bu)$_3$P (12%) and 1.7 mL (i-Pr)$_2$NH (2.4 equiv., 12 mmol) were injected into the tube successfully, and the mixture was stirred vigorously. Then a solution of 1.56 g p-ethynylbenzaldehyde (2.4 equiv., 12 mmol) in 6 mL 1,4-dioxane was injected into the reaction mixture in 2 h. The mixture was maintained at RT overnight and the progress was monitored by thin layer chromatography. Upon completion, 15 mL ethyl acetate (EA) was added and the mixture was filtered through a pad of silica gel with copious EA rinsings. The filtrate was concentrated and the crude product was purified via recrystallization from hexane/$CHCl_3$. The pure product was isolated as a bright yellow solid in 70% yield. ^1H NMR (400 MHz, d_6-acetone) δ 10.08 (s, 2H), 7.98 (d, J = 8.4 Hz, 2H), 7.74 (d, J = 8.2 Hz, 2H), 4.45 (s, 4H). ^{13}C NMR (100 MHz, d_6-acetone) δ 192.17, 145.73, 137.07, 132.55, 130.45, 129.17, 99.94, 96.89, 84.18, 65.92. MALDI-TOF m/z calcd. for $C_{24}H_{14}O_4S$ 398.0613 $[M]^+$, found 398.0532.

epy. In a round bottom flask, 0.398 g compound **2** (1.0 equiv., 1.0 mmol) was added and the flask was degassed and refilled with N_2 for 3 times. Then 10 mL absolute EtOH was added and the mixture was stirred and brought to 60 °C. 2-Ethoxyethanol was added in a dropwise manner until the solid was fully dissolved. To the resulting solution, 0.5 mL 2-acetylpyridine (4.0 equiv., 4.0 mmol) was injected and the solution was stirred for 10 min. After that, 0.308 g KOH (4.7 equiv., 4.7 mmol) was added in one go and 5.8 mL 28% $NH_3 \cdot H_2O$ (4.3 equiv., 4.3 mmol) was injected subsequently. The mixture was kept at 95 °C for 15 h and then the reaction was maintained in air for 2 days. After the mixture was cooled to RT, it was filtered and the residue was washed with deionized water until the filtrate was neutral. The crude product was purified via recrystallization from MeOH/$CHCl_3$, which furnished the pure product as a dark yellow solid in 37% yield. ^1H NMR (400 MHz, d_8-THF) δ 8.89 (s, 4H), 8.78 – 8.61 (m, 8H), 7.99 (d, J = 8.4 Hz, 4H), 7.91 (td, J = 7.7, 1.8 Hz, 4H), 7.69 (d, J = 8.4 Hz, 4H), 7.43 – 7.32 (td, J = 7.7, 1.8 Hz, 4H), 4.36 (s, 4H). ^{13}C NMR (100 MHz, d_8-THF) δ 157.17, 156.88, 150.03, 149.77, 145.21, 139.36, 137.50, 132.72, 128.01, 124.75, 121.67, 119.03, 100.16, 97.08, 82.33, 65.89. MALDI-TOF m/z calcd. for $C_{52}H_{32}N_6O_2S$ 805.2380 $[M+H]^+$, found 805.2413.

Metallopolymers PM(epy). As a general procedure, 19 mg **epy** (24 μmol) was dissolved in 10 mL THF under nitrogen. With vigorous stirring, a solution of 24 μmol $M(BF_4)_2$ in a mixed solvent of 0.5 mL deionized water and 2 mL MeOH was added into the ligand solution in a dropwise manner. The reaction was maintained at RT for 1 day, after which the precipitate was collected by filtration, washed thoroughly with deionized water and THF, and dried in a vacuum oven. Pure **PCo(epy)**, **PNi(epy)** and **PCu(epy)** were furnished as a red powder, a dark yellow powder and a brown powder, respectively. The elemental analysis data were calculated against the polymer structure in which every terminal terpyridine site coordinated with one metal ion and the coordination number of every metal terminal metal ion was satisfied by chelating three water molecules.

PCo(epy). A red powder. FTIR (KBr): 1569 cm^{-1} (C=N of pyridine). Elemental analysis calcd. for $(C_{52}H_{32}N_6O_2SB_2F_8Co)_4Co(BF_4)_2(H_2O)_6$: C, 55.63; H, 3.14; N, 7.49; S, 2.86. Found: C, 55.54; H, 3.58; N, 7.00; S, 3.17.

PNi(epy). A dark yellow powder. FTIR (KBr): 1569 cm^{-1} (C=N of pyridine). Elemental analysis calcd. for $(C_{52}H_{32}N_6O_2SB_2F_8Co)_{11}Co(BF_4)_2(H_2O)_6$: C, 58.47; H, 3.12; N, 7.87; S, 3.00. Found: C, 58.78; H, 3.63; N, 7.39; S, 3.18.

PCu(epy). A brown powder. FTIR (KBr): 1569 cm^{-1} (C=N of pyridine). Elemental analysis calcd. for $(C_{52}H_{32}N_6O_2SB_2F_8Co)_2Co(BF_4)_2(H_2O)_6$: C, 51.42; H, 3.15; N, 6.92; S, 2.64. Found: C, 51.13; H, 3.54; N, 6.45; S, 3.08.

2. XPS

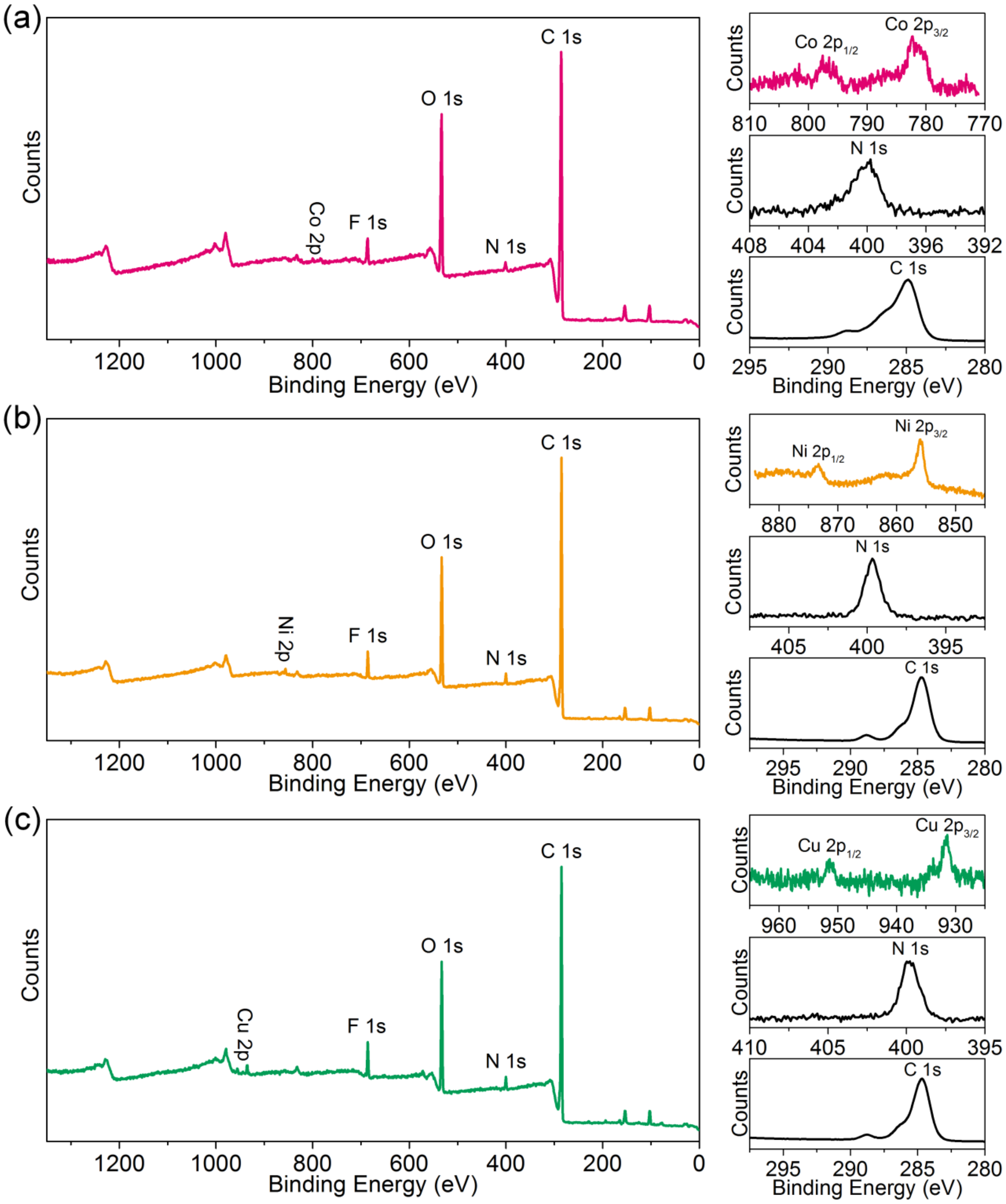

Figure S2. (Left panel) XPS survey spectra of (a) **PCo(epy)**, (b) **PNi(epy)** and (c) **PCu(epy)** and (right panel) the corresponding high-resolution spectra focusing on the metal, nitrogen and carbon elements.

3. NMR Spectra

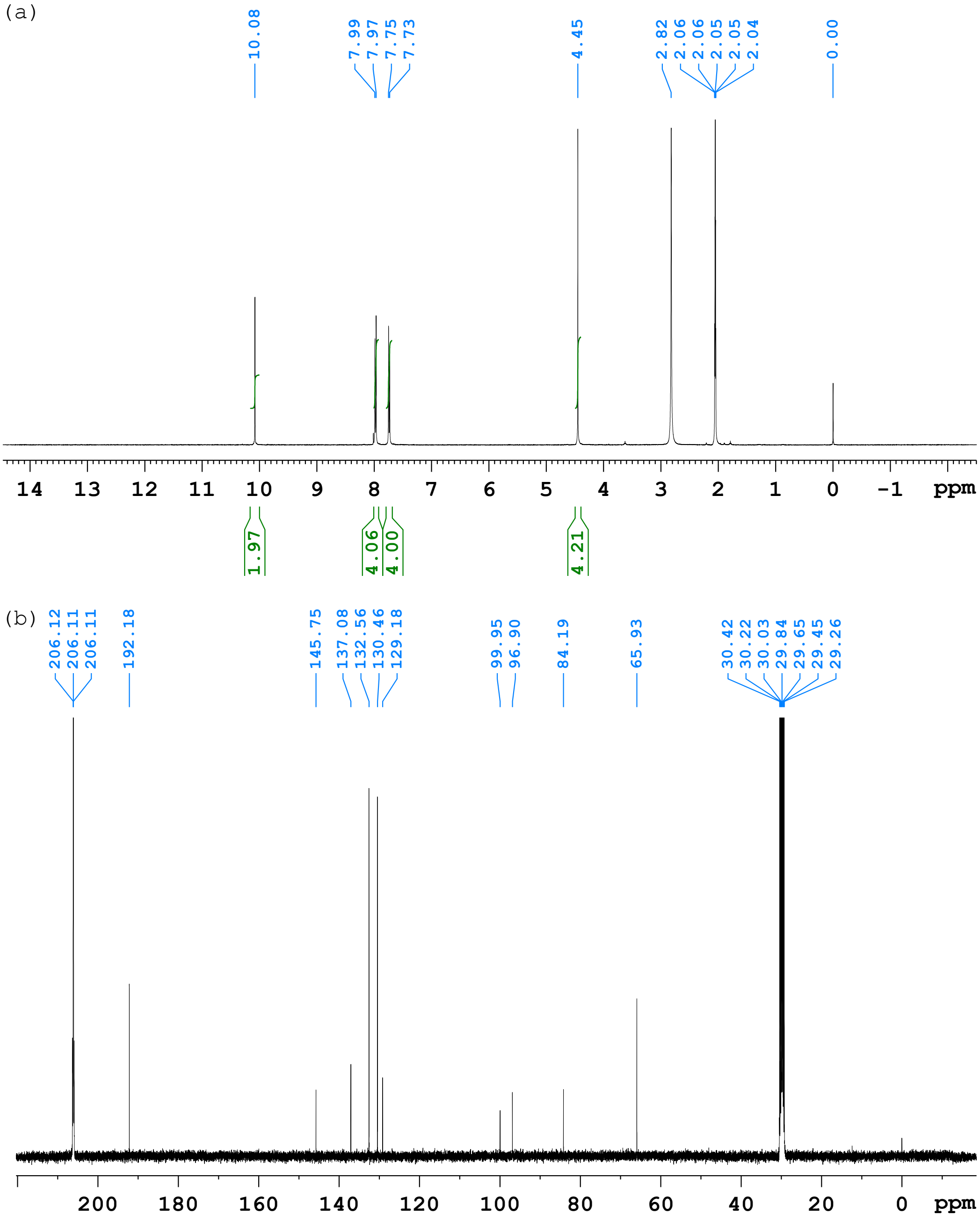

Figure S3. (a) ^{1}H (400 MHz) and (b) ^{13}C NMR (100 MHz) spectra of compound **2** in d_6-acetone.

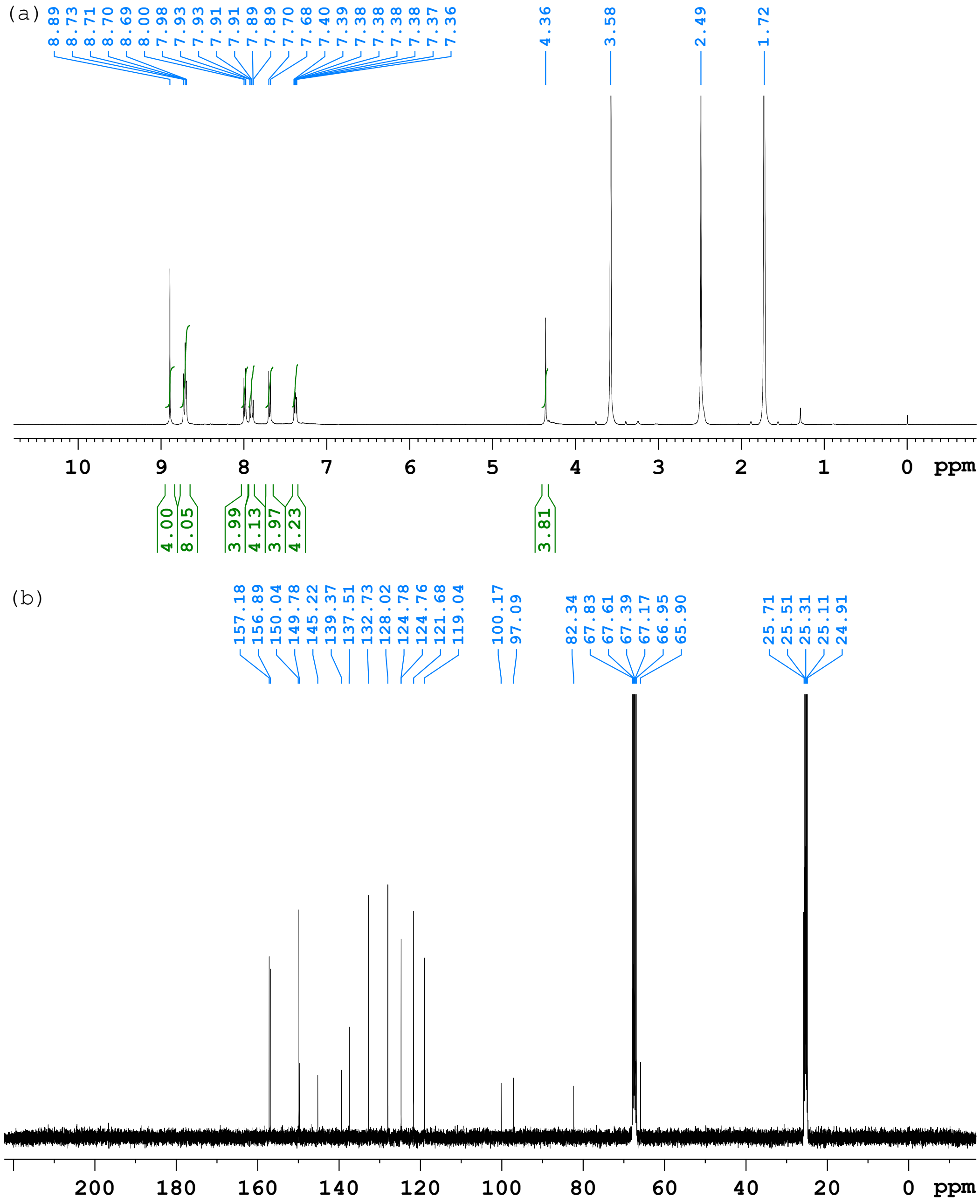

Figure S4. (a) ^{1}H (400 MHz) and (b) ^{13}C NMR (100 MHz) spectra of **epy** in d_8-THF.

4. Mass Spectra

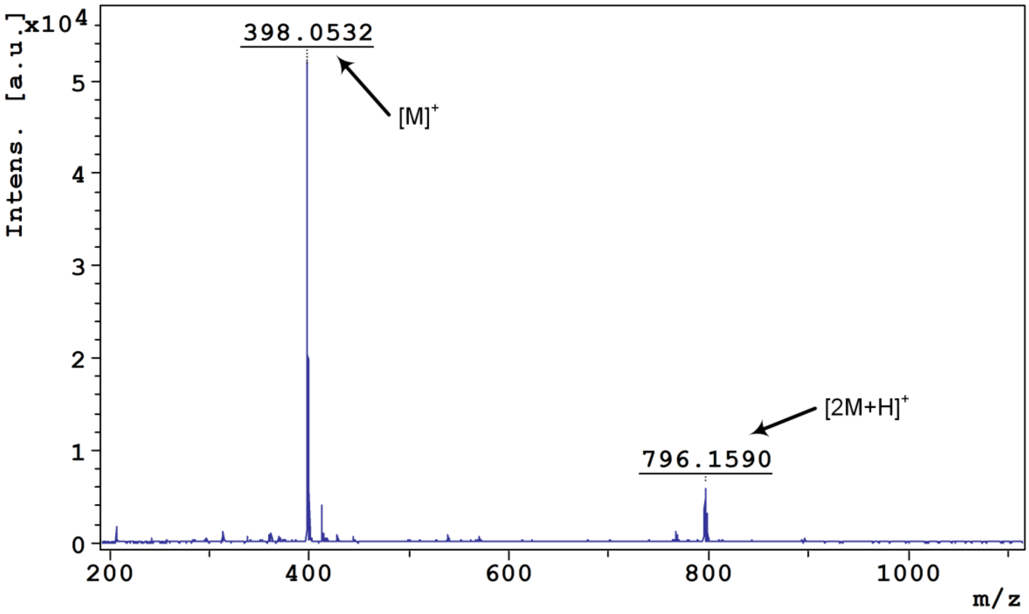

Figure S5. MALDI-TOF mass spectrum of compound **2**.

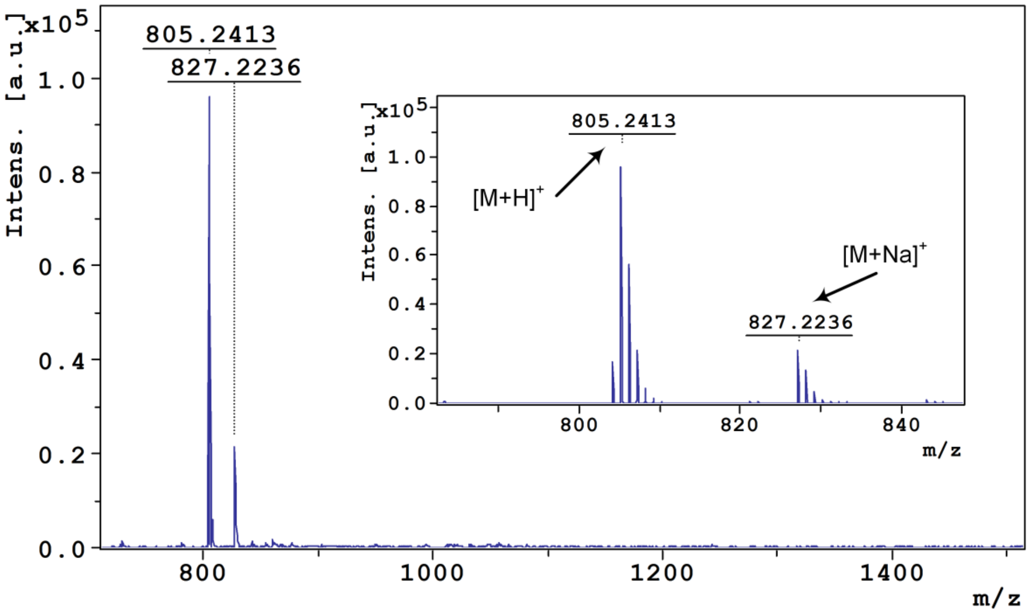

Figure S6. MALDI-TOF mass spectrum of **epy**.

5. UV-Vis-NIR absorption spectrum of SWCNT

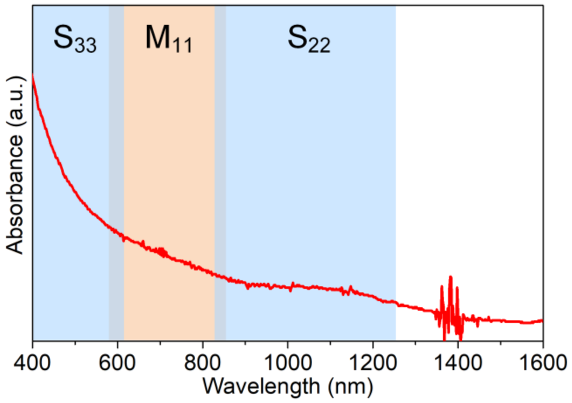

Figure S7. UV-Vis-NIR spectrum of SWCNT in the mixture of *o*-dichlorobenzene and toluene (1:2, *V*:*V*).

As displayed in Figure S7, the characteristic absorption bands corresponding to the semiconducting SWCNT (S_{22} and S_{33}) and the metallic SWCNT (M_{11}) [2] were detected, suggesting the co-existence of the SWCNT in both types.

6. TEM image of SWCNT

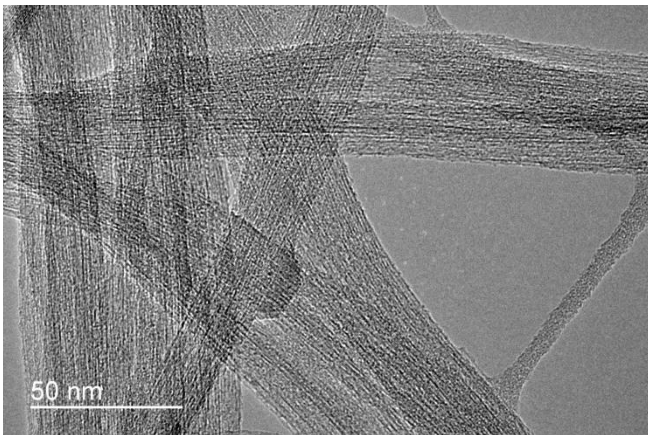

Figure S8. TEM image of the SWCNT bundles.

Figure S8 revealed the morphology of the CNT used in our experiments. A typical microstructure of the SWCNT bundles [3] were observed, which was formed by the aggregation of individual SWCNT.

References

1. Hundertmark, T.; Littke, A. F.; Buchwald, S. L.; Fu, G. C., $Pd(PhCN)_2Cl_2/P(t\text{-}Bu)_3$: A Versatile Catalyst for Sonogashira Reactions of Aryl Bromides at Room Temperature. *Org. Lett.* **2000**, *2*, 1729-1731.
2. Lei, T.; Pochorovski, I.; Bao, Z., Separation of Semiconducting Carbon Nanotubes for Flexible and Stretchable Electronics Using Polymer Removable Method. *Acc. Chem. Res.* **2017**, *50*, 1096-1104.
3. Zhang, Q.; Zhou, W.; Xia, X.; Li, K.; Zhang, N.; Wang, Y.; Xiao, Z.; Fan, Q.; Kauppinen, E. I.; Xie, S., Transparent and Freestanding Single-Walled Carbon Nanotube Films Synthesized Directly and Continuously via a Blown Aerosol Technique. *Adv. Mater.* **2020**, *32*, 2004277.
